# Supplementary material for: Evaluation of Genomic Prediction for Pasmo Resistance in Flax
Source: Int J Mol Sci. 2019 Jan 16;20(2):359. doi: 10.3390/ijms20020359 (PMC6359301; doi:10.3390/ijms20020359)
Supplement: Supplementary file 1 [file ijms-20-00359-s001.pdf]

International Journal of *Molecular Sciences*

MDPI

Article

Evaluation of Genomic Prediction for Pasmo Resistance in Flax

Liqiang He [1,2], Jin Xiao [2], Khalid Y. Rashid [3], Gaofeng Jia [4], Pingchuan Li [3], Zhen Yao [3], Xiue Wang [2], Sylvie Cloutier [1,*], and Frank M. You [1,2,*]

[1] Ottawa Research and Development Centre, Agriculture and Agri-Food Canada, Ottawa, ON K1A 0C6, Canada; liqiang.he@canada.ca (L.H.); sylvie.cloutier@canada.ca (S.C.); frank.you@canada.ca (F.M.Y.);
[2] State Key Laboratory of Crop Genetics and Germplasm Enhancement, College of Agriculture, Nanjing Agricultural University/JCIC-MCP, Nanjing Jiangsu210095, China; xiaojin@njau.edu.cn (J.X.); xiuew@njau.edu.cn (X.W.)
[3] Morden Research and Development Centre, Agriculture and Agri-Food Canada, Morden, MB R6M 1Y5, Canada; khalid.rashid@canada.ca (K.Y.R.); zhen.yao@canada.ca (Z.Y.); lipingchuan@gmail.com (P.L.)
[4] Crop Development Centre, University of Saskatchewan, Saskatoon, SK S7N 5A8, Canada; gaofeng.jia@usask.ca (G.J.);

* Correspondence: frank.you@canada.ca (F.M.Y.); sylvie.cloutier@canada.ca (S.C.);
Tel.: +1-613-759-1539 (F.M.Y.); +1-613-759-1744 (S.C.)

Supplementary figures and tables

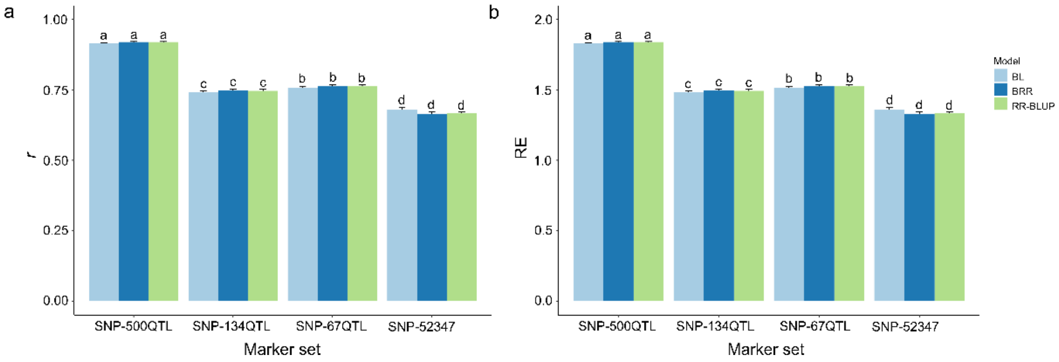

Figure S1. The accuracies (*r*) (**a**) and relative efficiency (*RE*) (**b**) of prediction models built with combinations of four marker sets and three statistical models—ridge regression best linear unbiased prediction (RR-BLUP), Bayesian LASSO (BL), and Bayesian ridge regression (BRR)—with average pasmo severity (PS) across five years (PS-mean dataset) using random five-fold cross-validation. The statistical significances of *r* among different statistical models are labeled with letters. Different letters show significant difference at a 5% probability level.

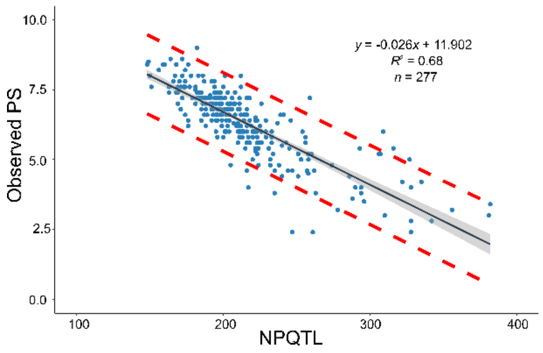

Figure S2. Linear regression of observed PS to the number of positive-effect QTL (NPQTL) in the 277 accessions that were used as a training population for genomic prediction. The grey band represents the 95% confidence interval. The red dashed lines represent upper and lower bounds of the 95% prediction interval. The average width of the prediction interval for all predicted values was 2.70.

Table S1. Analysis of variance of the accuracy (*r*) and relative efficiency (*RE*) for different marker sets and pasmo severity (PS) datasets using random five-fold cross-validation.

**Source of variance**	**Degree of freedom**	***r***			***RE***		
		MS	***F***	***P* value**	***MS***	***F***	***P* value**
Marker set (M)	3	237.8	51,955.8	<2.2e-16	951.1	51,955.8	<2.2e-16
PS dataset (P)	5	73.5	16,063.1	<2.2e-16	294.05	16,063.2	<2.2e-16
M × P	15	2.4	526.9	<2.2e-16	9.65	526.9	<2.2e-16
Residuals	60,186	0.005			0.02		

MS: mean square; *F*: *F* value.

Table S2. Genomic prediction (GP) of pasmo severity (PS) for 93 flax cultivars and breeding lines using the GP model built with SNP-500QTL and DS-mean of 277 accessions as training data.

Cultivars /Lines	Improvement status[1]	Observed PS[2]	Predicted PS by GP[3]	NPQTL[4]	Predicted PS by NPQTL[5]
CN100952	U	8.2 ± 1.1	8.6	152	7.9 (6.5 - 9.4)
CN101052	B	3.4 ± 0.5	3.6	329	3.3 (1.9 - 4.8)
CN101053	B	3.0 ± 0.7	3.3	359	2.6 (1.1 - 4.0)
CN101392	C	6.0 ± 1.9	5.9	230	5.9 (4.5 - 7.3)
CN18988	C	3.2 ± 1.1	3.8	346	2.9 (1.5 - 4.3)
CN98072	C	5.4 ± 2.8	6.1	245	5.5 (4.1 - 6.9)
CN100929	C	2.6 ± 0.5	3.1	356	2.6 (1.2 - 4.1)
CN18983	C	2.8 ± 0.8	3.4	350	2.8 (1.4 - 4.2)
CN19001	C	2.0 ± 0.0	3.0	354	2.7 (1.3 - 4.1)
CN97610	C	6.2 ± 1.1	6.2	211	6.4 (5.0 - 7.8)
CN97616	C	8.4 ± 0.9	7.7	179	7.2 (5.8 - 8.7)
CN101405	U	3.6 ± 0.5	3.7	305	4.0 (2.5 - 5.4)
CN101055	B	5.8 ± 1.6	4.8	261	5.1 (3.7 - 6.5)
CN101116	B	7.2 ± 1.5	6.8	216	6.3 (4.9 - 7.7)
CN101094	C	3.6 ± 1.5	4.3	311	3.8 (2.4 - 5.2)
CN35791	C	5.2 ± 2.2	5.1	267	5.0 (3.5 - 6.4)
CN97530	L	6.8 ± 1.5	6.7	192	6.9 (5.5 - 8.3)
CN97533	L	7.2 ± 1.6	7.0	171	7.5 (6.0 - 8.9)
CN101394	U	6.4 ± 2.7	5.2	265	5.0 (3.6 - 6.4)
CN101395	U	4.4 ± 2.3	4.6	311	3.8 (2.4 - 5.2)
CN101402	U	6.8 ± 1.9	7.1	199	6.7 (5.3 - 8.1)
CN101406	U	3.8 ± 1.3	4.1	288	4.4 (3.0 - 5.8)
CN101382	U	7.0 ± 1.2	7.0	203	6.6 (5.2 - 8.0)
CN32546	C	7.0 ± 2.1	6.7	217	6.3 (4.8 - 7.7)
CN33393	C	3.8 ± 1.6	3.7	367	2.4 (0.9 - 3.8)
CN97665	B	7.8 ± 1.3	8.0	145	8.1 (6.7 - 9.5)
CN98903	B	4.5 ± 1.7	4.9	263	5.1 (3.6 - 6.5)
CN98946	C	4.8 ± 1.1	5.6	252	5.3 (3.9 - 6.8)
CN101338	U	5.8 ± 0.8	6.4	212	6.4 (5.0 - 7.8)
CN97953	C	6.0 ± 1.6	6.3	201	6.7 (5.3 - 8.1)
CN101373	U	5.6 ± 1.5	5.9	222	6.1 (4.7 - 7.5)
CN101469	B	6.4 ± 0.5	6.6	205	6.6 (5.2 - 8.0)
CN101536	B	4.4 ± 1.5	5.3	217	6.3 (4.8 - 7.7)
CN101542	B	8.4 ± 0.5	7.6	183	7.1 (5.7 - 8.6)
CN101560	B	4.6 ± 2.6	5.1	260	5.1 (3.7 - 6.6)
CN101580	B	5.8 ± 1.6	5.7	216	6.3 (4.9 - 7.7)
CN101594	B	6.2 ± 1.3	6.1	195	6.8 (5.4 - 8.2)

CN18980	C	6.4 ± 2.6	5.5	224	6.1 (4.7 - 7.5)
CN19004	C	6.6 ± 2.3	6.9	213	6.4 (5.0 - 7.8)
CN19157	C	6.0 ± 1.0	6.3	203	6.6 (5.2 - 8.0)
CN33388	C	6.0 ± 0.7	5.5	245	5.5 (4.1 - 6.9)
CN97671	C	7.6 ± 1.1	7.9	177	7.3 (5.9 - 8.7)
Linola989	C	4.8 ± 1.7	5.4	255	5.3 (3.9 - 6.7)
PrairieBlue	C	5.8 ± 1.3	5.3	247	5.5 (4.1 - 6.9)
CN98683	C	6.0 ± 2.2	6.3	246	5.5 (4.1 - 6.9)
CN100881	C	7.2 ± 0.8	7.0	188	7.0 (5.6 - 8.4)
CN98475	C	6.4 ± 1.1	7.0	208	6.5 (5.1 - 7.9)
CN98712	C	6.8 ± 1.8	6.8	198	6.8 (5.3 - 8.2)
CN98767	C	7.2 ± 1.5	6.8	198	6.8 (5.3 - 8.2)
CN98773	C	5.4 ± 1.5	5.8	215	6.3 (4.9 - 7.7)
CN101367	U	1.8 ± 0.4	2.6	351	2.8 (1.3 - 4.2)
CN101325	U	7.0 ± 1.2	6.4	199	6.7 (5.3 - 8.1)
CN97300	C	7.2 ± 0.8	7.3	198	6.8 (5.3 - 8.2)
CN98275	C	7.0 ± 1.4	7.1	173	7.4 (6.0 - 8.8)
CN98278	C	6.4 ± 1.5	6.5	211	6.4 (5.0 - 7.8)
CN98854	C	7.4 ± 1.1	6.9	189	7.0 (5.6 - 8.4)
CN98250	C	7.0 ± 0.7	6.6	204	6.6 (5.2 - 8.0)
CN98254	C	7.0 ± 0.7	6.4	204	6.6 (5.2 - 8.0)
CN98440	C	6.0 ± 0.7	5.6	220	6.2 (4.8 - 7.6)
CN98468	C	7.0 ± 1.2	6.8	191	6.9 (5.5 - 8.3)
CN98974	C	6.6 ± 1.5	6.6	209	6.5 (5.1 - 7.9)
CN98240	L	5.3 ± 1.7	6.2	215	6.3 (4.9 - 7.7)
CN101310	U	7.4 ± 0.9	6.9	179	7.2 (5.8 - 8.7)
CN98569	U	6.4 ± 2.4	6.5	210	6.4 (5.0 - 7.8)
CN101240	B	6.4 ± 1.8	6.2	217	6.3 (4.8 - 7.7)
CN101026	B	5.4 ± 2.2	5.5	242	5.6 (4.2 - 7.0)
CN97064	C	5.4 ± 2.5	5.9	210	6.4 (5.0 - 7.8)
CN97092	C	5.2 ± 0.8	5.9	214	6.3 (4.9 - 7.7)
CN97103	C	5.0 ± 1.0	4.7	229	5.9 (4.5 - 7.4)
CN101289	B	4.4 ± 1.5	4.7	286	4.5 (3.0 - 5.9)
CN101299	B	3.0 ± 0.7	3.5	297	4.2 (2.8 - 5.6)
CN101307	B	7.0 ± 1.0	7.4	186	7.1 (5.7 - 8.5)
CN97487	C	7.2 ± 1.5	7.4	168	7.5 (6.1 - 8.9)
CN97520	C	5.6 ± 2.2	5.9	214	6.3 (4.9 - 7.7)
CN101375	U	7.0 ± 1.2	6.6	212	6.4 (5.0 - 7.8)
CN97147	C	8.2 ± 0.8	8.0	157	7.8 (6.4 - 9.2)
CN100837	U	6.0 ± 0.7	5.9	238	5.7 (4.3 - 7.1)
CN101332	U	7.2 ± 1.3	7.3	196	6.8 (5.4 - 8.2)
CN30861	C	6.6 ± 1.8	6.8	190	7.0 (5.6 - 8.4)

CN97404B	B	6.6 ± 1.3	7.0	191	6.9 (5.5 - 8.3)
CN33399	C	5.8 ± 1.6	6.3	237	5.7 (4.3 - 7.1)
CN97403	C	7.4 ± 1.3	7.2	172	7.4 (6.0 - 8.8)
CN97407	C	6.8 ± 2.3	6.1	208	6.5 (5.1 - 7.9)
CN97921	C	5.4 ± 1.3	6.1	227	6.0 (4.6 - 7.4)
CN98541	C	6.4 ± 1.5	5.9	214	6.3 (4.9 - 7.7)
CN98812	C	5.6 ± 1.7	6.4	251	5.4 (4.0 - 6.8)
CN97341	C	7.0 ± 1.0	6.5	196	6.8 (5.4 - 8.2)
CN101572	B	5.0 ± 1.4	4.9	243	5.6 (4.2 - 7.0)
CN101016	C	4.6 ± 0.9	5.2	228	6.0 (4.6 - 7.4)
CN100885	U	5.8 ± 1.5	5.9	233	5.8 (4.4 - 7.2)
CN100790	C	8.0 ± 0.7	7.3	187	7.0 (5.6 - 8.4)
CN97402	B	6.8 ± 1.6	6.5	194	6.9 (5.4 - 8.3)
CN98644	B	7.3 ± 1.0	7.1	194	6.9 (5.4 - 8.3)

[1] B: Breeding lines; C: Cultivars; L: Landrace; U: Unknown. [2] Average PS across five years (2012-2016) and their standard deviations. [3] Predicted PS based on the GP model built with the marker set SNP-500QTL, the PS-mean dataset of 277 accessions, and the statistical model RR-BLUP. [4] NPQTL: number of positive-effect QTL. [5] Predicted by NPQTL (the equation is $y = -0.026x + 11.902$ based on 277 accessions as training data). The values in parentheses are prediction intervals.
